# Analysis of the efficacy of MALDI-TOF MS technology in identifying microorganisms in cancer patients and oncology hospital environment

**DOI:** 10.1016/j.heliyon.2025.e42015

**Published:** 2025-01-19

**Authors:** Grażyna Czeszewska-Rosiak, Iwona Adamczyk, Agnieszka Ludwiczak, Piotr Fijałkowski, Paweł Fijałkowski, Magdalena Twarużek, Michał Złoch, Dorota Gabryś, Wioletta Miśta, Andrzej Tretyn, Paweł Piotr Pomastowski

**Affiliations:** a Centre for Modern Interdisciplinary Technologies, Nicolaus Copernicus University in Torun, Wilenska 4 Str., Torun 87-100, Poland; b Departnemt of Plant Physiology and Biotechnology, Nicolaus Copernicus University in Torun, Lwowska 1 Str., 87-100 Torun, Poland; c Department of Physiology and Toxicology, Kazimierz Wielki University, Chodkiewicza 30 Str., Bydgoszcz, Poland; d Department of Immunology, Faculty of Biological and Veterinary Sciences, Nicolaus Copernicus University in Torun, Lwowska 1 Str., 87-100 Toruń, Poland; e Department of Environmental Chemistry and Bioanalytics, Faculty of Chemistry, Nicolaus Copernicus University in Toruń, Gagarina 7 Str., 87-100 Toruń, Poland; f Radiotherapy Department, Maria Sklodowska-Curie National Research Institute of Oncology, Wybrzeze Armii Krajowej 15 Str., Poland

**Keywords:** MALDI-TOF MS, Microorganisms' identification, Pathogen detection, Opportunistic bacteria, Healthcare environments

## Abstract

Rapid diagnostics of microbes in hospitals are crucial for promptly identifying infections, enabling timely and appropriate treatment. The study was conducted to evaluate the effectiveness of the matrix-assisted laser desorption ionization time-of-flight mass spectrometry (MALDI TOF MS) technology in the microbial profiling of hospital environments and patient samples. The objective was to determine the microbial landscape in swabs collected from hospitalized patients and their immediate environments, using MALDI to compare the capabilities of two systems: BRUKER and ZYBIO. The analysis resulted in 1012 microbial identifications from patient samples (N = 81), encompassing 96 species, and 1496 identifications from hospital surface samples (N = 108), covering 124 species. Predominantly identified microorganisms in patients’ samples included *Staphylococcus epidermidis, Staphylococcus aureus, Staphylococcus capitis, Steptococcus salivarius*, and *Micrococcus luteus*, whereas environmental samples chiefly yielded *S. epidermidis, Staphylococcus hominis, Staphylococcus warneri*, and *Microcccus luteus*. 33 species were found in both types of samples, highlighting a significant microbial interchange within hospital settings. Both MALDI systems showed high consistency in results at both genus and species levels. Nevertheless, mismatches in identification between both MALDI systems were noted, particularly within *Brevibacterium, Streptococcus, Bacillus, Staphylococcus*, and *Neisseria* genera. This study presents the precision of MALDI technology in microbial identification and highlights the need for ongoing enhancements, especially in the expansion and updating of databases, to bolster its diagnostic effectiveness further.

## Highlights

• MALDI-TOF MS proves useful for rapid and precise microbial identification.
• Both BRUKER and ZYBIO are effective in microbiological ID.
• Opportunistic bacteria identified in both patient samples and hospital surroundings.
• Continuous improvement needed for MALDI-TOF MS databases to enhance accuracy.

## 1 Introduction

Bacteria are omnipresent, thriving in virtually all environments. The public health perspective underscores the significance of microbiological safety within patient accommodations, notably in doctors' offices and hospitals [1,2]. Clinics, serving as the nexus for individuals often with weakened immune systems, elevate the risk of transmitting different types of infections. The World Health Organization (WHO) has highlighted hospital-acquired infections as a critical concern, emphasizing the microbiological safety of patients in healthcare settings as crucial for safeguarding patient health and life, especially in the context of oncology care for patients treated with radiotherapy and chemotherapy [[3], [4], [5], [6]]. An important element of infection control is rapid and accurate microbiological diagnosis as part of screening and infection diagnosis. The speed, accuracy, and precision of the obtained results are crucial elements of patient treatment. Gram staining and antigen tests allow to obtain the results in less than an hour. However, these methods are not very sensitive or precise [7]. Routine methods based on microbiological culture and biochemical identification are time-consuming and often insensitive, and thus targeted therapy is delayed, which reduces the success of curing patients. Molecular methods such as polymerase chain reaction (PCR) are highly sensitive and complement conventional methods. It is an excellent tool for screening diagnostics of patients for viruses or multidrug-resistant bacteria, as well as in cases of suspected specific infections (e.g., MRSA [methicyllin-resistant *Staphylococcus aureus*], KPC [*Klebsiella pneumoniae* carbapenemase], *Legionella pneumophila*, influenza, RSV [respiratory syncytial virus], SARS-CoV-2 [severe acute respiratory syndrome coronavirus 2]) [8,9]. However, it does not provide a complete pattern of the potential microbiological threat in a given location. Identification through 16S rRNA sequencing and Next-Generation Sequencing (NGS) delivers excellent identification results and allows for the study of relationships between individual strains. However, both methods are time-consuming, labor-intensive, and require specialized equipment and well-trained personnel [10,11].

The matrix-assisted laser desorption ionization time-of-flight mass spectrometry (MALDI-TOF MS) technology appears to be a promising method, as rapid, accurate and cost-effective tool for microbial identification. In recent years, the MALDI TOF MS method has revolutionized clinical microbiology. Its simplicity of use, speed of identification, and the relatively small number of bacteria required for identification have made this technique a fundamental diagnostic tool in microbiological laboratories worldwide, simultaneously displacing biochemical methods [[12], [13], [14], [15]]. Since 2009, the Maldi Biotyper system has obtained the CE mark associated with the European IVD system (in vitro diagnostic products) [16]. This technique has been developed since the 1970s. Furthermore, in the late 1990s, the use of α-cyano-4-hydroxycinnamic acid as a matrix allowed for direct and rapid identification without additional preparation of the examined colony. The technique is based on the analysis of protein composition (primarily ribosomal proteins) using MALDI TOF mass spectrometry. During the analysis, the laser ionizes the proteins, and the time required for the ionized proteins to travel to the detector is determined as the mass-to-charge ratio (*m/z*) of the examined proteins. The signal intensity is recorded as a spectrum, which is compared with reference spectra for bacterial identification [13,17,18].

Identification capabilities of MALDI TOF MS are continually being developed with new solutions, such as rapid and direct diagnostics of cultured microorganisms, including sepsis from a positive blood bottle (SEPSI-TYPER) or urine sample, which significantly increases the chances of quick and appropriate therapy for the infected patient [19,20]. Additionally, MALDI-TOF facilitates precise and rapid identification of conventional microbiological cultures, providing a broad overview of the tested material, especially those rich in bacteria [9,21,22]. Researching the capabilities of bacteria identification and improving the MALDI-TOF method may also prove to be an excellent tool not only for identification but also for assessing antibiotic resistance (e.g., ESBL - Extended-Spectrum Beta-Lactamases) The advantages of employing MALDI TOF MS technology enable the implementation of quicker and more accurate treatments, thereby enhancing the patient's prospects for recovery [[22], [23], [24], [25], [26]].

The aim of this study was to present the microbiological profiles of swabs collected from different parts of the body of oncology patients, as well as from their immediate surroundings, and to perform a comprehensive statistical evaluation of the obtained data. In addition, the study undertakes a comparative analysis of two state-of-the-art MALDI-TOF MS technologies—BRUKER and ZYBIO—assessing their efficiency and precision in characterizing the microbiological composition of clinical samples.

## 2 Materials and methods

### 2.1 Research material, ethics and consent

Swab samples were collected at the Maria Skłodowska-Curie National Research Institute of Oncology in Gliwice, Poland, between March 15 and March 22, 2023. To preserve their integrity, the samples were immediately frozen and stored at −80 °C until the time of analysis. The study encompassed a comprehensive examination of 189 samples, categorically divided into two distinct groups for a thorough analysis. The first group comprised 81 samples were derived from 45 hospitalized oncological patients, collected from a range of body sites such as the throat (31 samples), ears (27), nose (26), and various sections of masks (29), offering insights into the microbiota directly associated with patients. The second group consisted of 108 samples from various hospital surfaces, including phones (31 samples), keyboards (20), water dispensers (12), door handles (10), air conditioners (7), and patients' cabin seats (7), among others, thereby providing a comprehensive snapshot of potential microbial reservoirs. The procurement of patient samples was conducted under the highest ethical standards, with informed consent obtained from each participant prior to collection, thereby ensuring the ethical integrity of the research. Ethical approval for this study was granted by the Ethics Committee of the National Institute of Oncology (NIO-PIB), under the approval number KB/430-78/22. The study was designed and executed in strict adherence to the principles of Good Clinical Practice, with all patients providing written informed consent, underscoring the study's commitment to ethical research practices and the protection of patient rights and welfare. The criteria for exclusion from the experiment are the patient's lack of consent to have a sample taken for testing as well as pregnant women and incapacitated persons. This careful and ethical approach to sample collection not only ensured the reliability and validity of the research findings but also reinforced the study's dedication to advancing scientific knowledge while respecting the dignity and rights of all participants.

### 2.2 Isolation and cultivation

For throat swabs, dilutions were standardized to 10^−2^ and 10^−3^, while samples from other sources underwent dilutions in a range of 10^−1^ to 10^−2^, according to the sample type and expected microbial load. All dilutions were performed using sterile physiological saline solution. A volume of 100 μL from each diluted sample was plated onto six distinct types of culture media, each selected for its ability to support the growth of specific groups of microorganisms. These media included Columbia Blood Agar Base (BLA) and Azide Blood Agar Base (AZI), both supplemented with 5 % defibrinated sheep blood and sourced from Oxoid, Basingstoke, United Kingdom, for the cultivation of a wide range of bacteria, including fastidious species. Schaedler Agar (SCH) from Merck, Darmstadt, Germany, was employed for the growth of anaerobic bacteria. MacConkey Agar (MC) was utilized to differentiate lactose-fermenting from non-lactose-fermenting enteric bacteria. VRE Agar Base (VRE), also from Oxoid, was specifically chosen for its efficacy in isolating *Enterococcus* species resistant to vancomycin. Lastly, Tryptic Soy Agar (TSA) from Sigma Aldrich, Steinheim, Germany, was used for the cultivation of a broad spectrum of bacteria. Following inoculation, the media were incubated for 48 h to allow for optimal bacterial growth. Aerobic cultures were maintained at 37 °C in atmospheric conditions conducive to the growth of aerobes, while anaerobic cultures were incubated at 37 °C in an environment with 5 % CO_2_ to cater to the needs of anaerobic bacteria.

### 2.3 Preparing samples for MALDI TOF MS analysis

Initially, a microbial colony was collected using a sterile disposable loop and placed onto a designated spot on the MALDI 96 target polished steel BC plate. This sample was then evenly spread within the confines of the target spot to form a homogenous thin layer. Over this layer, 1 μL of 70 % formic acid (FA) solution was applied to aid in protein extraction and allowed to air dry at ambient temperature. Following this drying phase, the sample spots were coated with 1 μL of α-Cyano-4-hydroxycinnamic acid (α-CHCA) matrix solution, prepared in a solvent mix comprising 47.5 % HPLC grade water, 2.5 % trifluoroacetic acid, and 50 % acetonitrile, achieving a concentration of 10 mg/ml. Calibration of the system was conducted using specific standards: for the BRUKER system, 1 μL of the BRUKER Bacterial Test Standard (BTS) containing a synthetic extract of *Escherichia coli* DH5 alpha that provides a distinctive peptide and protein profile in the MALDI-TOF mass range of 3.6–17 kDa; for the Zybio system, a Microbiology Calibrator containing *E. coli* ATCC 25922 protein extract, alongside ribonuclease and myoglobin, covering the same mass range.

The bacterial colonies prepared for identification on the MALDI plate spots were identified using both devices—BRUKER and ZYBIO—in a randomly selected order.

### 2.4 Identification of microorganisms via the BRUKER MALDI biotyper platform (BRUKER)

The prepared samples on the targets were subjected to analysis using the microflex LT/SH MALDI-TOF mass spectrometer by BRUKER Daltonics GmbH, Bremen, Germany, which utilizes a nitrogen UV laser in positive ion mode. Spectrum acquisition was carried out manually utilizing the flexControl version 4.1 software, adhering to parameters outlined in foundational research. Post-acquisition, spectra underwent Savitzky-Golay smoothing, baseline adjustment via the TopHat algorithm, and calibration using the BTS standard in a quadratic mode, employing the flexAnalysis version 4.1 software. For ensuring robust identification, each sample was analyzed a minimum of five times. The accumulated spectra were used to identify microorganisms through MALDI Biotyper automation control and Bruker Biotyper 4.1 software and library (version rev. H/2021), which contains 10,834 entries (Bruker Daltonics).

### 2.5 Identification of microorganisms via the ZYBIO EXS 2600 platform (ZYBIO)

Parallel analyses were conducted on the ZYBIO EXS 2600 system equipped similarly with a nitrogen UV laser in positive ion mode, following the ZYBIO procedural guidelines. This process involved automatic spectrum collection and subsequent identification leveraging the EX-Accuspec v1.0.21.7 software. Prior to identification, spectra calibration was performed using the ZYBIO Microbiology Calibrator to ensure precision. Reproducibility and reliability of the results were guaranteed by measuring each sample in at least five replicates, facilitating a comprehensive and comparative evaluation of the microbial isolates across both MALDI TOF MS platforms.

### 2.6 Identification of bacterial isolates using 16S rDNA sequencing

For the justification of the selected mismatched identifications, the sequencing of the 16S rRNA region was performed using the total bacterial genomic DNA isolated using E.Z.N.A.® Bacterial DNA Kit (Omega Bio-tek, Norcross, US), universal bacterial primers 27F (5-AGAGTTTGATCMTGGCTCAG-3) and 1492R (5-GGTTACCTTGTTACGACTT-3), thermostable Taq DNA polymerase (Qiagen, Hilden, Germany) and Mastercycler pro S thermocycler (Eppendorf AG, Hamburg, Germany) used for amplification of the 16S rRNA region via PCR technique according to the procedure described in our earlier work [27]. PCR products were sequenced via the Sanger dideoxy method in Genomed (Warsaw, Poland). Contigs were assembled via BioEdit Sequences Alignment Editor ver. 7.2.5 [28], and consensus sequences were compared with references sequences in rRNA/ITS databases of the National Center for Biotechnology Information via the BLAST algorithm (https://blast.ncbi.nlm.nih.gov/Blast.cgi?PAGE_TYPE=BlastSearch).

### 2.7 Statistical analysis

The statistical analysis in this study was designed to ensure the reliability and validity of our findings regarding the microbial profiling of hospital environments and patient samples using MALDI TOF MS technology. Employing the advanced capabilities of the PS IMAGO PRO 9.0 package (IBM SPSS Statistics), it was applied statistical methodologies to assess the significance of our results and the performance differences between the BRUKER and ZYBIO systems.

Statistical significance (*p* < 0.05) was determined using the Student's t-test. These *p*-values were denoted directly on the graphical representations of our data, with asterisks used to indicate the level of statistical significance: *∗p* < 0.05 signifying a statistically significant difference and *∗∗p* < 0.01 indicating a highly significant difference. To visually present and facilitate the interpretation of our data, we employed various graphical tools: (i) Histograms were used to depict the distribution of microbial identifications across different sample sources, providing an immediate visual representation of the microbial diversity and prevalence; (ii) Two-dimensional scatter plots offered insights into the comparative accuracy and reliability of the BRUKER and ZYBIO systems by displaying the distribution of identification scores. This graphical representation was pivotal in highlighting the performance efficacy of each system, and (iii) Pie charts were adeptly utilized to represent the proportion of identification mismatches between the two systems, effectively summarizing the challenges encountered in microbial identification.

## 3 Results

**Fig. 1:**
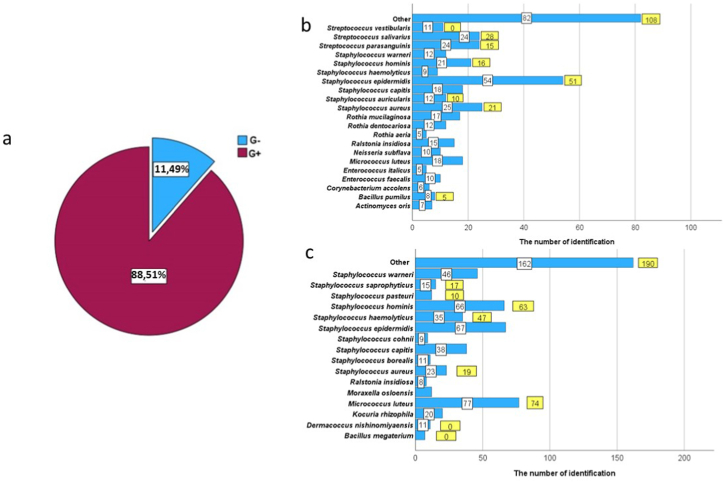
Bacterial Composition and Gram Staining Classification Across Analyzed Specimens. a) distribution of Gram-positive (G+) and Gram-negative (G-) bacterial populations within the examined specimens, b) taxonomic identification of bacterial species derived from patient swabs (yellow labels indicate the quantity of statistically significant identifications by ZYBIO, white labels indicate BRUKER identifications), c) taxonomic distribution of bacterial species isolated from hospital environments. Species constituting less than 1 % of the total identified population are aggregated into an "Other" Category to enhance chart legibility.

This comprehensive study undertook an analysis of 189 samples to elucidate the microbial landscape within a hospital setting, encompassing both patient-related (N = 81) and environmental samples (N = 108) (Fig. S1 a, b). The analysis yielded a rich diversity of the microbiota, identifying 96 distinct species of bacteria and fungi from patient samples, with an additional two identifications at the genus level, averaging approximately 4.63 species per patient sample. This diversity underscores the complex microbial ecosystems present on patients and their immediate contact surfaces. Environmental samples from the hospital setting revealed even greater diversity, with 124 different species of bacteria and fungi identified, including one at the genus level, resulting in an average of 5.63 species per environmental sample. This finding not only highlights the vast array of microbial species present in hospital environments but also reflects on the potential for cross-contamination between these environments and the patients residing within them. A significant portion of the identified microorganisms, constituting 88.51 % of all tested species, were Gram-positive, encompassing 128 different species. The remaining species were Gram-negative, 58 species in total (Fig. 1 a). In patient swabs, the dominant species were *Staphylococcus epidermidis* (N = 51, 13,74 %), *Staphylococcus aureus* (N = 25, 6,36 %) *Streptococcus salivarius* (N = 24, 6,10 %), *Streptococcus parasanguinis* (N = 24, 6,10 %), *Staphylococcus hominis* (N = 21, 5,34 %), *Staphylococcus capitis* (N = 18, 4,58 %) and *Micrococcus luteus* (N = 18, 4,58 %), where 'N' indicating the frequency of bacteria occurrence identified by the BRUKER system. The most common bacteria from hospital surfaces represented the species *M. luteus* (N = 77, 12,66 %), *S. epidermidis* (N = 67, 11,02 %), *S. hominis* (N = 66, 10,86 %), *Staphylococcus warneri* (N = 46, 7,57 %), *S. capitis* (N = 38, 6,25 %), *Staphylococcus haemoliticus* (N = 35, 5,76 %), *S. aureus* (N = 23, 3,78 %). (Fig. 1b and c).

**Fig. 2:**
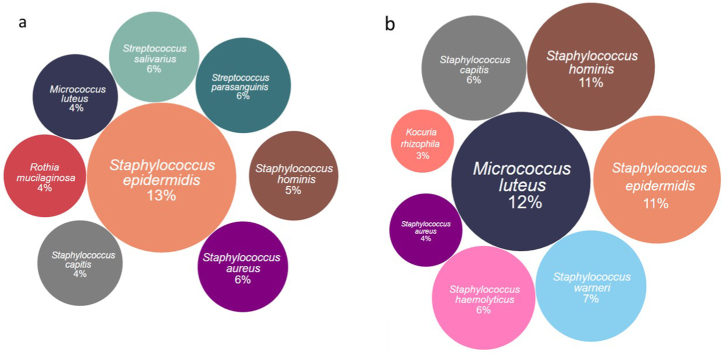
Quantitative Distribution of Eight Predominant Bacterial Species Isolated from Patient (a) and Hospital Environments (b) indicated by occurrence percentage in collected samples. The diameter of each circle is proportional to the relative abundance of corresponding bacterial species within the analyzed samples. Chromatic variation distinguishes among diverse bacterial species.

The distribution of individual bacterial species in the samples is illustrated in Fig. 2. In patients' samples, the predominant species was S. epidermidis, accounting for 13 % and for hospital surface – *M. luteus*, 12 %. The lower variation in the most dominant bacteria percentage can be observed for patients’ samples were the share of the other most frequently isolated species did not exceed 6 %. For the hospital environment, the share of the subsequent most common species ranged from 11 % for S. epidermidis and S. hominis to 3 % and 4 % for Kocuria rhizophila and *S. aureus*, respectively (Fig. 2a and b).

**Fig. 3:**
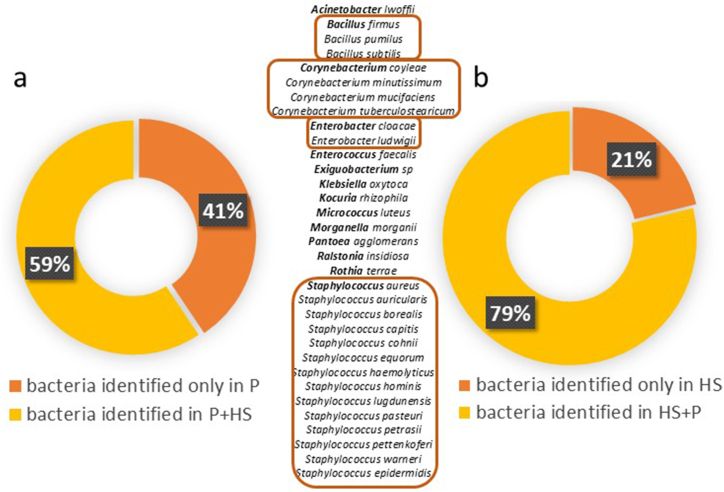
The Proportion of High-confidents Identification of Unique and Coincident Bacteria Collected from Patient (P) (a) and Hospital Environment (HS) (b) with 33 bacteria Species Identified in Both Type of Samples. The same genus of bacteria identified in P and HS samples are presented as list. Identification results of the Bruker system.

In both types of samples, 33 species were identified as the common species constituting 34.34 % of the species in patient samples and 26.61 % in environmental samples (list on Fig. 3). Considering the frequency of occurrence in all analyzed samples, these common species accounted for 59 % of the species identified in patient samples that were also detected in the patient's environment. Conversely, 79 % of the species identified in environmental samples were also present in analyzed body part of the patients (Fig. 3).

**Fig. 4:**
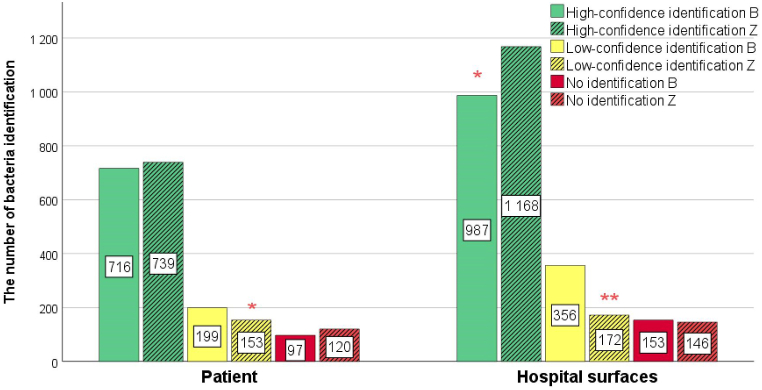
Comparative Analysis of Microorganism Identifications via BRUKER (B) and ZYBIO (Z) Systems from Hospital Surfaces and Patient Samples. Statistical significance between B and Z identification was assessed using Student's t-test, with p-values indicated by red asterisks: ∗*p* < 0.05, ∗∗*p* < 0.01.

The MALDI-TOF MS identified 1496 bacterial isolates from environmental swabs and 1012 from patient swabs. Identifications were classified as high-confidence (score ≥2.00), low-confidence (score 1.70–1.99), or no identification (score <1,70), with high-confidence referring to species-level and low-confidence to genus-level. A comparison between the BRUKER and ZYBIO systems indicated differences in the degree of confidence. From patients’ swabs, BRUKER and ZYBIO systems achieved similar high-confidence identifications (716 (70.75 %) and 739 (73.02 %), respectively). BRUKER system allows to identify statistically significant more bacteria at genus level (199 (19.66 %) and 153 (15.12 %) for BRUKER and ZYBIO system respectively). 97 (9.58 %) and 120 (11.86 %) no identification were obtained for BRUKER and ZYBIO system, respectively (Fig. 4). From hospital surfaces, BRUKER system achieved 987 (65.98 %) high-confidence identifications versus 1168 (78.04 %) by the ZYBIO system, 356 (23.80 %) low-confidence identifications versus 172 (11.50 %) by the ZYBIO system, and 153 (10.23 %) no identification by BRUKER versus 146 (9.76 %) by the ZYBIO system. 97 (9.58 %) and 120 (11.86 %) no identification were obtained for BRUKER and ZYBIO system, respectively (Fig. 4).

**Fig. 5:**
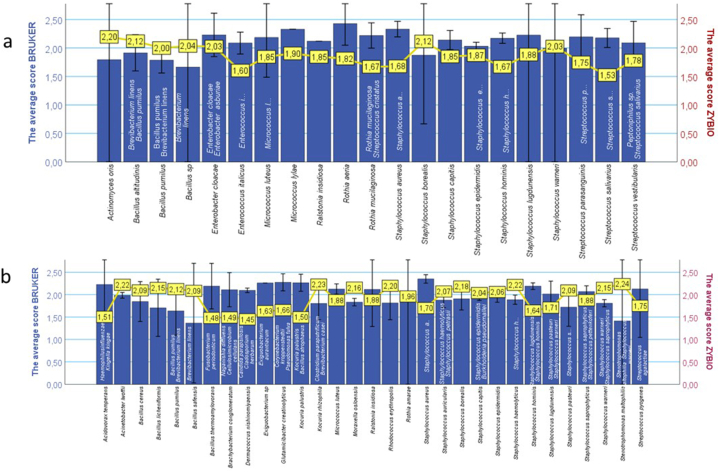
Variability in MALDI-TOF MS Identification Across Sample Types: (a) Patient Samples and (b) Hospital Environment. ZYBIO Identification Scores Are Highlighted in Yellow, with a Corresponding Yellow Line Illustrating the Trend in Score Fluctuations. Alternative species to BRUKER identification were denoted on the bar chart columns. To enhance chart clarity, only the most prevalent bacterial species were presented. Detailed descriptions of bacterial species identified exclusively by ZYBIO are provided in the Supplementary Materials.

Fig. 5 indicate the differential score values and consequent mismatches in microbial identification between BRUKER and ZYBIO systems. The twenty-one discrepancies from patient sample were detected. Six discrepancies were related to the cases where ZYBIO system reported scores greater than 2.00 (species-level confidence), while BRUKER provided only IDs at genus-level confidence (1.70–1.99) or lack of identification (<1.70). Fifteen discrepancies show score >2.00 in the case of BRUKER, whereas ZYBIO demonstrates confidence only at the genus level or lack of identification (Fig. 5 a). Thirty-one identification differences from hospital environment samples were noticed, of which ZYBIO identified sixteen with a score greater than 2.00, while BRUKER provided an identification with score less than 2.00. For fifteen discrepancies with scores greater than 2.00 (BRUKER), the ZYBIO system assigned score below 2.00 (Fig. 5 b).

Regardless of the type of the samples, the most significant differences in the number of mismatch identifications were obtained for the genus *Staphylococcus*. The largest number of the mismatches of the ZYBIO system identification compare with BRUKER were noticed in the case of *S. aureus* for hospital surface samples and *S. epidermidis*, *S. hominis*, and *S. salivarius* collected from patient samples (Fig. 5 a). The ZYBIO system identified six different alternative species for all the species mentioned. For both types of samples, a consistent trend in the identification of *Bacillus pumilus* by BRUKER was observed. The ZYBIO system identified these species as *B. pumilus* and *Brevibacterium linens*. Interesting, depending on the sample type and MALDI system, we obtained different identification results for the same species. For example, *M. luteus* for hospital surface samples was identified by two systems as *M. luteus* (Fig. 5 b). However, the same species collected from patient samples and identified by BRUKER as *M. luteus* were identified by the ZYBIO system as *M. luteus*, *Clostridium paraputrificum*, and *Brevibacterium casei*. Similar observations were noticed for *S. epidermidis*. Regarding the samples collected from the patient, the ZYBIO system indicates no unambiguous identification of *S. epidermidis* as *Staphylococcus epidermidis*, *Kingella denitrificans*, *Aspergillus niger*, *Actinomyces odontolyticus*, *Talaromyces rugulosus*, and *Staphylococcus saccharolyticus*.

**Fig. 6:**
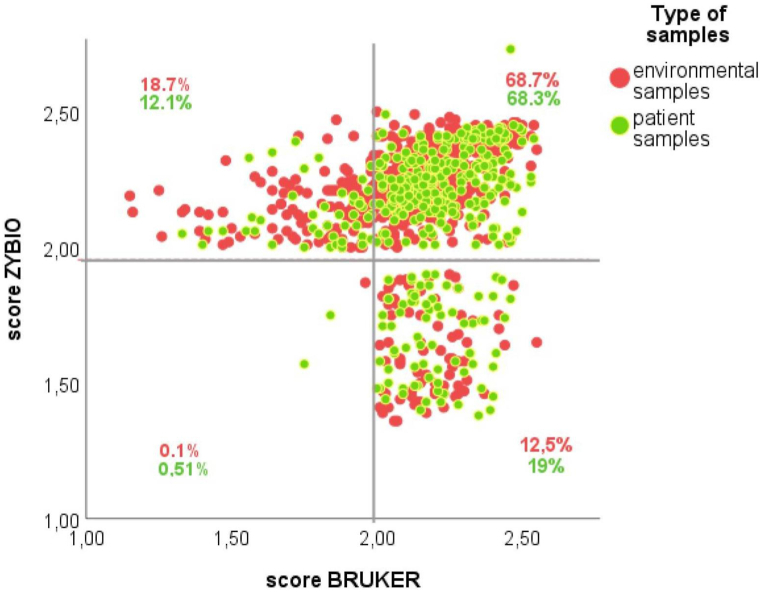
Two-Dimensional Scatter Plot Illustrating the Comparative Distribution of Identification Scores from BRUKER to ZYBIO. The percentage values assigned to four parts of the chart reflecting the quality of identification. Samples derived from hospital and environmental sources are represented as red and green points, respectively.

The quality and distribution of scores from BRUKER and ZYBIO systems are presented as a scatter plot (Fig. 6). Regardless of the system, high-confidence identifications were achieved for 68.3 % and 68.7 % of the patient and hospital surface samples, respectively. 12.1 % of patient samples and 18.7 % of hospital surface samples were identified with a higher confidence level (≥2.00) by the ZYBIO system. In comparison, another 12.5 % of hospital surface samples and 19 % of patient samples were identified with a score ≥2.00 for BRUKER and ≤2.00 for ZYBIO simultaneously.

**Fig. 7:**
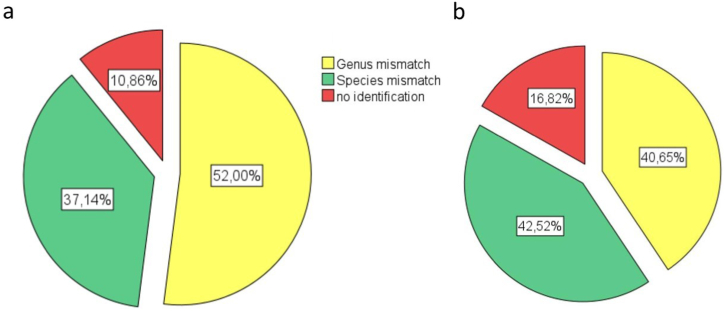
The pie charts demonstrate the level of overall mismatch in patient (a) and hospital environmental samples (b).

The significant difference in the type of mismatch was recognized between two types of samples (Fig. 7). Total of 405 discrepancies were observed, with 190 (45 %) with patient-derived samples and 215 (55 %) with environmental samples. 52 % and 41 % of the genus mismatch were noticed for patience and hospital samples, respectively. Species mismatch (37 % and 42 %) and no identification (10 % and 16 %) were obtained for patience and hospital samples, respectively (Fig. 7).

The most significant species mismatch identification in patient samples involved identification of *B. linens* (N = 18) by ZYBIO as five different species of *Bacillus* (*B. altitudinis* (N = 4), *B. pumilus (*N = 7), and *B. species* (N = 5), *Lactobacillus paralimentarius* (N = 1) and *Lysobacter xinjiangensis* (N = 1)) by BRUKER. Another interesting case was identification of *Streptococcus salivarius* by BRUKER. ZYBIO identified these species as seven different species representing seven different genera, including fungal species: *Aspergillus fumigatus* and *Cladosporium cladosporioides* (Fig. S2 b). Species mismatches were also noted for hospital environmental samples. The genus of *Bacillus* (identified by BRUKER) was clearly identified as *B. linens* by ZYBIO. Another mismatch included *Glutamicibacter creatinolyticus* (identified by BRUKER) and recognized by ZYBIO as *Corynebacterium kroppenstedtii*, and another mismatch identified pairs *Cytobacillus oceanisediminis* (BRUKER) – *Bacillus firmus* (ZYBIO) (current name according to LPSN is *Cytobacillus firmus*), *Staphylococcus saprophyticus* (BRUKER) – *Mycobacterium senegalensis* (ZYBIO), and the pair *S. borealis* (BRUKER) – *S. haemolyticus* (ZYBIO) (Fig. S2 a).

*Neisseria* showed identification genus mismatch in patient samples (Fig. S2 d). BRUKER distinguishing individual strains (e.g., *N. perflava, N. flavescens*), while ZYBIO grouped *Neisseria* species into two results: *N. flava/flavescens/subflava/perflava* and *N. mucosa/macacae/sicca*. A significant genus mismatch in environmental samples is represented for the pair *S. borealis* (BRUKER) vs. *S. haemoliticus* (ZYBIO) (N = 16). Another important mismatch is the genus designation of *Micrococcus* spp. by the ZYBIO system, while BRUKER reports these identifications as *M. luteus* (N = 11) (Fig. S2 c).

Additionally, the BRUKER system does not collect spectra of sufficient quality automatically, resulting in the no bacteria identification. This problem occurred nineteen and thirty-six times in the case of patient and hospital samples, respectively. Interestingly, ZYBIO identified bacteria from these samples, often with a score exceeding 2.0. More detailed information regarding identification discrepancies was provided in Fig. S2.

To resolve discrepancies in the identification of deposited strains, 14 microorganisms were selected, which during testing exhibited high identification scores in MALDI-TOF MS methods, and were subsequently analyzed using 16S rRNA gene sequencing. 16S rRNA sequencing confirmed the identification of nine out of the fourteen strains compared using the BRUKER system, whereas for the remaining five strains, identification could not be definitively resolved even with this method. Detailed identification results are presented in Table S1.

## 4 Discussion

Most bacterial species in human-inhabited environments exhibit similarities to the microbiota associated with humans. This correlation is widely recognized and represents a significant epidemiological discovery, particularly in patient care environments. Our study reveals the detection of bacteria in 100 % of samples collected from high-touch surfaces and patient swabs. In this study, the most frequently occurring genus was *Staphylococus* identified in almost 57 % of the analyzed samples from the hospital surfaces and 41.5 % from patients. The prevalence of this bacterial genus in environments frequented by patients and medical staff stay is given its close association with humans. The study of Alvarez et al. presented a similar distribution to our results (69 % *Staphylococcaceae* and 89 % Gram-positive bacteria form nasal swabs) [29]. Bacteria composition of Intensive Care Units (ICU) surfaces was represented mostly by *Staphylococcus* (31 %), *Propionibacterium* (13 %), *Pseudomonas* (13 %), *Bacillus* (10 %), *Enterococcus* (7 %), *Streptococcus* (5 %), and *Acinetobacter* (7 %) [30]. A different result of microbiome composition describes Gong et al. where the most dominant were *Streptococcus* genus as a result of microbiome identification of the throat of health patients. Throat has a specific microbiota, which differs from the rest of the body. The study by Chung et al. indicated the isolation of Gram-positive cocci in 36 % of the tested clinical samples and a high percentage of Gram-negative bacilli (45.6 %) [31]. The difference in our findings, compared to those reported by Chung, may result from the nature of the studied samples. Chung examined samples from the hospital-acquired infections. Our study involved swabs from patients residing in hospital settings, where the main health problem of patients was cancer, not bacterial infection. Additionally, geographical differences may also contribute to the differences observed.

The study found that 33 species were common to both patients and hospital surfaces, accounting for 59 % of all identifications in patients and 79 % of those from the patient environment. These results suggest a potential flow of bacteria between individuals and their environment, which could significantly impact the epidemiological situation in the hospital, particularly when opportunistic bacteria are present among the environmental isolates. Many pathogenic bacteria can survive on surfaces for extended periods of time, from several hours to several months depending on the type of bacteria and the environmental conditions. For example, *A. baumannii* and *E. faecium* on variable materials can remain viable at least four weeks, *S. aureus* remained viable for at least one week [32,33].

Fortunately, the presence of bacteria on surfaces do not automatically lead to infection, but they can be an important factor in the transmission of infection. Infection requires the successful transfer of sufficient numbers of viable bacteria to a susceptible host. Touching contaminated surfaces can lead to infection if bacteria are transferred to mucous membranes (e.g. eyes, nose, mouth) or broken skin, there is sufficient number of bacteria present, the condition of the surface are growth friendly e.g. nonporous of not e.g. porous [34]. Some bacteria can form biofilms on surfaces, which not only increases their survival but also makes them more resistant to disinfectants and antibiotics [32]. The risk is particularly high in soldiers who are more susceptible to the effects of weakened immune systems and invasive procedures, such as the study patients. However, in this analysis, neither phenotyping nor molecular characterization of strains was performed, so it is not possible to definitively determine the scale of microorganism transmission between the environment and humans and vice versa. Among the commonly identified species, this study also revealed the presence of opportunistic microorganisms considered clinically important in the context of the epidemiology of infections in hospitalized patients, such as *Enterobacter cloacae, Enterococcus faecalis, Klebsiella oxytoca, Morganella morganii, Staphylococcus aureus, Staphylococcus haemolyticus* and *Staphylococcus epidermidis*. Nevertheless, there is considerable evidence of microbes being transmitted from humans to the environment and from the environment to humans. There are reports presenting partial dependencies between microorganisms inhabiting humans and their surroundings. Based on these findings, one can estimate the scale, factors, and mechanisms influencing the spread of microorganisms. Undoubtedly, the composition of the human microbiome is related to social and environmental factors. Microorganisms transmitted within society affect a wide range of processes related to human health, including the risk of transmitting infectious diseases [35]. Individuals living together share 12 % of their gut microbiome and 32 % of their oral microbiome [36]. This is particularly significant in the context of the spread of microorganisms in the hospital environment, where patients often stay in multi-bed rooms and medical staff care for multiple patients simultaneously. Tannhäuser investigated the contamination of phone surfaces among medical staff before and during the COVID-19 pandemic. 99.3 % of the smartphones presented the clinically significant bacteria on their surfaces (*S. aureus, Enterococcus* spp.*, Enterobacterales*). Another type of threat is posed by the ventilation and air conditioning systems inside buildings and vehicles. The microbiologically contaminated filters in these systems can also be a source of bacteria and fungi in human environments [37]. These studies confirmed possibility of expansion of microorganisms between patient and their surrounding environment. This dynamic bilateral flow poses a potential threat to public health, emphasizing the need for stringent infection control measures and ongoing surveillance to mitigate the spread of infectious agents, particularly those resistant to conventional treatments [38].

Some microorganisms are commonly present in the external environment and the human body. However, despite their familiar presence on the human body, some are seldom found in the living environment. An example of this is *Streptococcus* and *Neisseria*. This study, *Streptococcus* spp. was identified in 50 (61.73 %), while *Neisseria* spp. was identified in 14 (17.28 %) of the patient samples. None of the 108 samples collected from the patient's surroundings yielded cultured of these genera. This may result from their limited ability to survive outside the human body, high sensitivity to external conditions, and high nutritional requirements. The enriched media and the stable temperature with microaerophilic conditions are necessary to achieve growth of *Streptococcus* and *Neisseria* [39]. Johani identified the *Streptococcus* genus in 9 % of the environmental samples tested, with no indication of the *Neisseria* genus [30]. This underscores the challenge of sustaining these bacteria outside their natural human host environment, highlighting their specific ecological niches and survival strategies.

Developing an effective, rapid, and precise method to identify the microbiological composition of the environment and patient samples is essential to preventing hospital infections. There are increasing reports indicating that MALDI TOF MS technology is displacing other microorganism identification methods, including biochemical and molecular methods [12,40]. This is due to the high efficiency of the MALDI TOF MS method, as confirmed by numerous comparative studies indicating an identification accuracy ranging from 86.4 % to even 100 %, depending on the type of study and the strains tested [[41], [42], [43], [44], [45], [46]]. An example study conducted by van Veen presents a comparative performance of identification using MALDI TOF MS and biochemical methods, in relation to 16S rRNA. This prospective study included 980 clinical isolates of bacteria and yeasts. Correct identification to the species level was 92.2 % and 83.1 %, respectively [45]. However, what has been observed is that MALDI TOF MS technology has limitations. Comparative analysis between two distinct systems employing MALDI TOF MS technology has uncovered a mismatch in identifying specific microorganisms. This indicates variability in the performance of MALDI TOF MS platforms, which may impact the reliability of microbial identification in clinical diagnostics [45,46]. The discrepancies in the score values between the BRUKER and ZYBIO systems can be attributed to different methods of generating reference spectra with their respective databases. In the BRUKER's system, a signal must be replicated in at least 25 % of instances to qualify as a reference signal. The ZYBIO system sets this threshold at 50 % [26].

The BRUKER system encountered difficulties in accurately identifying bacteria from the *Bacillus* genus, with the average identification score consistently below 2.00 for both types of samples. In contrast, the ZYBIO system consistently exhibited an average score equal to or higher than 2.00 for all *Bacillus* spp. The study by Sibińska et al. showed a similar result, where the ZYBIO system identified a greater number of Bacillus spp. at the species level [26]. Similarly, the study by Caldeira confirms the identification difficulties within the Bacillus genus, even when using the 16S method [47]. These results highlight the variability in performance between different MALDI TOF MS systems, particularly in identifying specific bacterial genera, and underscore the importance of system selection based on the microbial identification needs of the clinical laboratory.

In the case of discrepancies in identification between MALDI systems, several factors can be considered. As mentioned earlier, there are inherent differences in how reference spectrum databases are constructed across these systems. Additionally, geographic factors could contribute to inconsistencies. The BRUKER, a European company, constructed reference spectra based on reference strain and bacterial strains from European territory. Conversely, ZYBIO is a Chinese company whose database was built on bacterial strains from the Chinese region. Bacteria worldwide are exposed to varying environmental factors, including temperature differences, leading to distinct defense mechanisms and potentially differing protein profiles during identification. Often, differences arise among closely related bacteria within the same genus; for instance, significant discrepancies exist between *Staphylococcus* strains and between *Enterococcus italicus* and *Enterococcus saccharolyticus* what was also observed in our study. However, the most intriguing case is the *Neisseria* genus. The reason for ZYBIO's mode of assigning results may be the close genetic relationship and highly similar protein profiles of *Neisseria* species, sometimes categorized into a common biovar. There are also instances where both MALDI TOF MS systems correctly identify a microorganism but utilize synonymous names, leading to apparent discrepancies in the results like for the identification of *Agrobacterium radiobacter* (BRUKER) – *Rhizobium radiobacter* (ZYBIO). Furthermore, the unreliability of identification, characterized by scores falling below the threshold of 1.70, represents another significant concern [48].

Moreover, previous studies have noted issues with correct identification of *Streptococcus mitis/oralis* by the BRUKER system, and present study also encountered some discrepancies [49,50]. The issues of accurate identification by MALDI-TOF MS is also evident for *Enterobacter cloacae/asburiae* [51]. The revision of the selected mismatch identification using results of the 16S rRNA sequencing showed that in most cases (∼64 %) Bruker identification was correct. Only in one case, Neisseria macacae/mucosa/sicca, the results of the Zybio identification can be considered as more correct since it more closely reflects the high similarity of the mentioned three strains, which does not allow a clear identification of any of the listed species. The use of the 16S rRNA sequencing revealed that identification *Neisseria* species using MALDI technique could be challenging since in another example of ID mismatch both systems demonstrated wrong identification of the *N. perflava* as *N. subflava* (Bruker) and *Neisseria flava/flavescens/subflava/perflava* (Zybio). Although Zybio indicated the possibility that the isolate is *N. perflava,* however, we considered it as wrong ID since the analysis of the 16S rDNA clearly separate both species – more than 1 % differences in nucleotides sequences. Similar problem is reported in the literature where researchers met a problem misidentification of non-pathogenic *Neisseria* species (eg. *N. subflava*, *N. cinerea*) as *N. meningitidis* during MALDI-TOF MS testing [52,53]. As possible accusation of this misidentification problem authors pointed the horizontal DNA transfers in *Neisser*ia genus and/or the insufficient number of non-pathogenic *Neisseria* spp. in the MALDI Biotyper library. Another group of mismatch identifications consist of spore-forming bacterial species belonged to such genera as *Bacillus, Cytobacillus* or *Paenibacillus*. The problem of the identification within this group could be associated with the spores production which was thoroughly examined in the work of Janiszewska et al. [54]. We also noted that in some cases the 16S rRNA gene sequence is not sufficient to differentiate the bacteria due to its high conservation as it is shown for *S. borealis* and *S. heamolyticus* (99.86–99.93 %) [55], *Neisseria macacae/mucosa/sicca* (more than 99.9 % homology) [56], *Streptococcus salivarius/vestibularis* (with a difference in 1–3 base values) [57], *Bacillus cereus* group (4–9 nucleotides variation) [58] or *Staphylococcus saprophyticus/pseudoxylosus* (99.9 % homology) [59]. In such cases, it is not easy to decide which system has demonstrated correct identification without analyzing other housekeeping genes such as sodA, rpoB, dnaJ, or tuf. Nevertheless, in some of the mentioned cases, the literature reports indicate the superiority of the MALDI technique over the 16S rRNA sequencing, as it was shown by Król et al. 2023, who revealed that the use of a new Bruker Biotyper library (MBT Compass Library Revision K), introduced in 2022, allowed for the correct recognition of the great majority (91.7 %) of bovine *S. borealis* isolates tested. Since our study used an older version of the Bruker Biotyper library (revision H from 2021), we cannot be sure about the superiority of the Bruker system over the Zybio regarding *S. borealis*/S. heamolyticus identification.The obtained results highlight the complexity of microbial identification using MALDI TOF MS technology and underscore the importance of understanding the nuances of each system, including database composition, naming conventions, and the interpretation of identification scores. Awareness of these factors is essential for clinicians and laboratory personnel to accurately interpret MALDI TOF MS results and make informed decisions regarding patient care. Furthermore, in cases of identification difficulties, the MALDI TOF MS system allows the use of external spectral databases and the expansion of the database with self-generated spectra, thereby enhancing the overall spectral database [60]. Moreover, MALDI technology enables rapid and precise identification of cultured colonies. Therefore, in the process of scientific or clinical research based on MALDI TOF MS technology, the uncultured part of the microbiome can be omitted.

## 5 Conclusions

Rapid and accurate identification of microorganisms is a critical component in maintaining the microbiological safety of the hospital environment. The use of MALDI-TOF-MS technology enables such identification capabilities. Furthermore, a comparative analysis of MALDI-TOF-MS technologies—BRUKER and ZYBIO—demonstrated that both systems are effective in microbial identification. However, further research is necessary to expand reference databases to enhance the diagnostic accuracy of MALDI-TOF-MS technology.

## CRediT authorship contribution statement

**Grażyna Czeszewska-Rosiak:** Conceptualization. **Iwona Adamczyk:** Writing – original draft, Methodology, Investigation, Formal analysis, Data curation, Conceptualization. **Agnieszka Ludwiczak:** Writing – review & editing, Writing – original draft, Software, Formal analysis, Conceptualization. **Piotr Fijałkowski:** Writing – original draft, Methodology, Investigation, Data curation. **Paweł Fijałkowski:** Writing – original draft, Methodology, Investigation, Data curation. **Magdalena Twarużek:** Supervision. **Michał Złoch:** Writing – review & editing, Validation, Supervision. **Dorota Gabryś:** Resources. **Wioletta Miśta:** Resources. **Andrzej Tretyn:** Supervision, Resources. **Paweł Piotr Pomastowski:** Writing – review & editing, Project administration, Funding acquisition.

## Data availability statement

The datasets generated during and/or analyzed during the current study are available in the RepOD repository, https://doi.org/10.18150/JXTCSC.

## Declarations ethical statement

The study was conducted in accordance with the Declaration of Helsinki and approved by the Institutional Ethics Committee of NIO-PIB Gliwice, Poland (protocol code KB/430-78/22 and date of approval 19 December 2019), and conducted in accordance with the principles of Good Clinical Practice. Each participant gave informed consent to participate in the study.

## Funding

This work was supported by 10.13039/501100005632The National Centre for Research and Development grant TANGO-V-A/0014/2021-00. Michał Złoch and Paweł Pomastowski is a members of Toruń Center of Excellence “Towards Personalized Medicine” operating under Excellence Initiative-Research University.

## Declaration of competing interest

The authors declare that they have no known competing financial interests or personal relationships that could have appeared to influence the work reported in this paper.
