# Supplementary material for: Analysis of the efficacy of MALDI-TOF MS technology in identifying microorganisms in cancer patients and oncology hospital environment
Source: Heliyon. 2025 Jan 19;11(2):e42015. doi: 10.1016/j.heliyon.2025.e42015 (PMC11791110; doi:10.1016/j.heliyon.2025.e42015)
Supplement: Multimedia component 1 [file mmc1.docx]

Supplementary Information


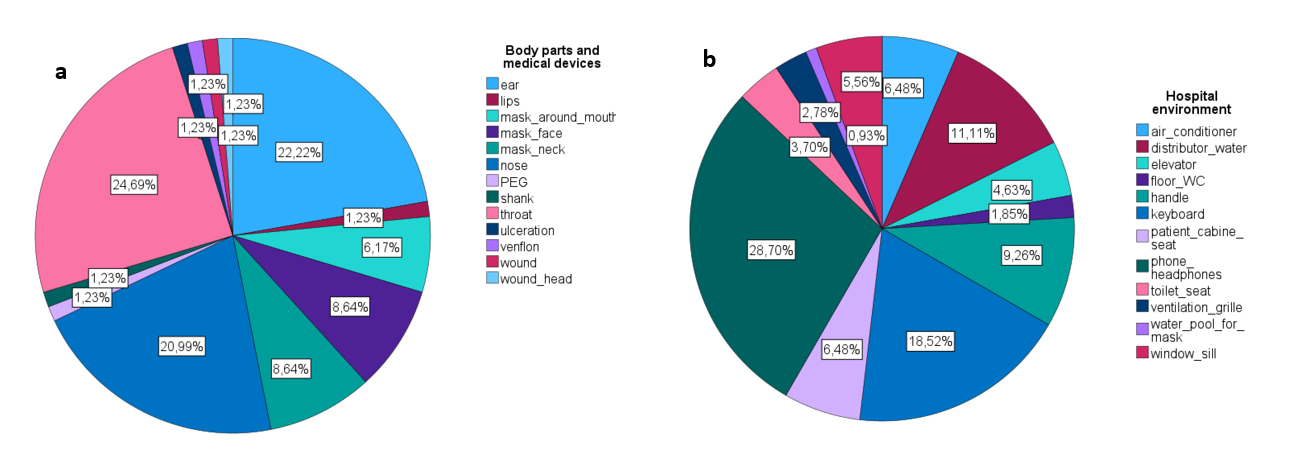


Supplementary Figure 1. The type of the tested samples: **a)** body parts of the patient and medical devices from which the swab was taken, **b)** hospital environment.

**The additional description to the Figures 6.**

“*Staphylococcus* a…” () was indicated by ZYBIO as: *Staphylococcus aureus*, *Staphylococcus simiae*, *Peptostreptococcus anaerobius*, *Staphylococcus equorum*, *Staphylococcus saprophyticus*, *Staphylococcus epidermidis*. “*Staphylococcus* h…” was indicated by ZYBIO as *Staphylococcus hominis*, *Bacillus smithii*, *Bacillus infantis*, *Nocardia farcinica*, *Microbacterium foliorum*. “*Staphylococcus* s…” was indicated by ZYBIO as *Staphylococcus saprophyticus*, *Mycobacterium senegalense*, *Staphylococcus haemolyticus*, *Staphylococcus pettenkoferi*. “*Enterococcus* i…” was indicated by ZYBIO as *Lactococcus lactis*, *Enterococcus saccharolyticus*, *Staphylococcus epidermidis*, *Streptococcus pyogenes*, *Vagococcus fluvialis*, “ *Micrococcus* l…” was indicated by ZYBIO as *Micrococcus luteus*, *Clostridium paraputrificum*, *Brevibacterium casei*. “Staphylococcus a…” was indicated by ZYBIO as *Staphylococcus aureus*, *Staphylococcus saprophyticus*, *Staphylococcus epidermidis*. “*Staphylococcus* e…” was indicated by ZYBIO as *Staphylococcus epidermidis, Kingella denitrificans, Aspergillus niger, Actinomyces odontolyticus, Talaromyces rugulosus, Staphylococcus saccharolyticus*, “*Staphylococcus* h…” was indicated by ZYBIO as *Staphylococcus hominis, Rothia dentocariosa, Rhodococcus rhodnii, Corynebacterium xerosis, Staphylococcus cohnii, Staphylococcus gallinarum*. “*Streptococcus* p…” was indicated by ZYBIO as *Streptococcus parasanguinis, Streptococcus salivarius, Streptococcus gallolyticus*. “*Streptococcus* s…” was indicated by ZYBIO as *Enterobacter gergoviae*, *Streptococcus gordonii, Acinetobacter pittii, Peptoniphilus sp., Dermatophilus congolensi, Streptococcus dysgalactiae*.


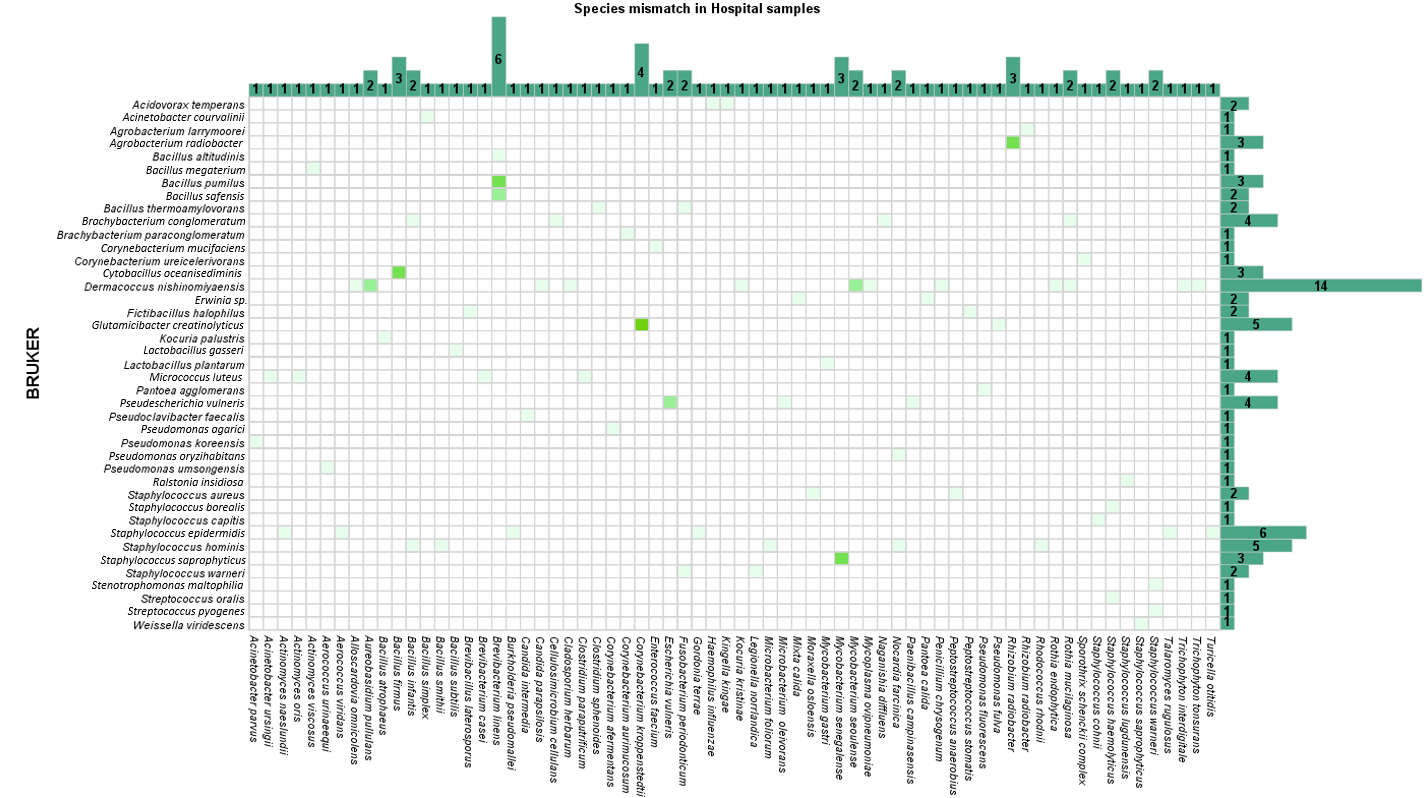


**ZYBIO**

**a**


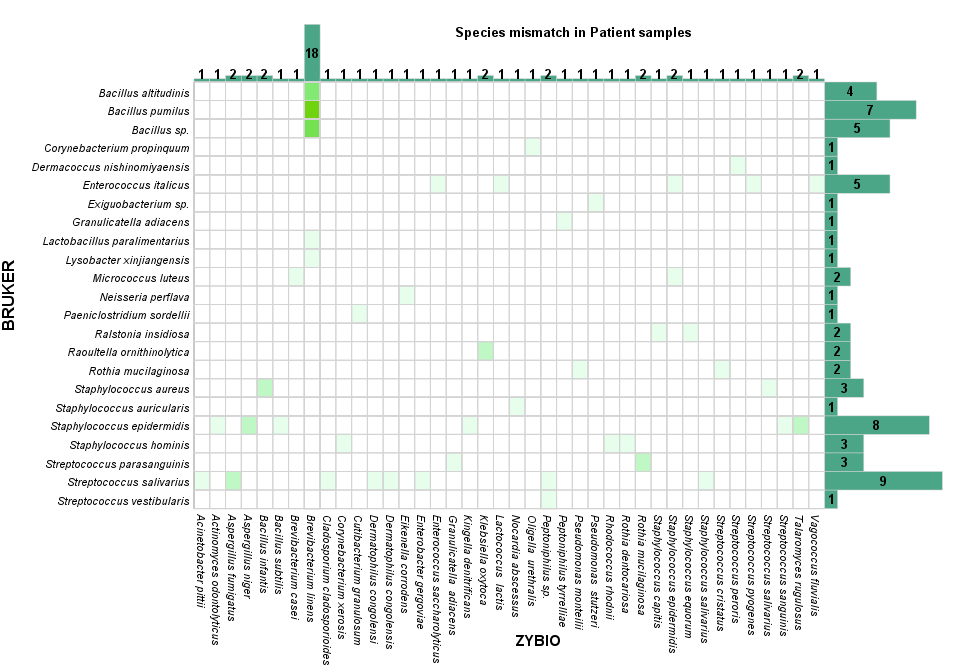


**b**


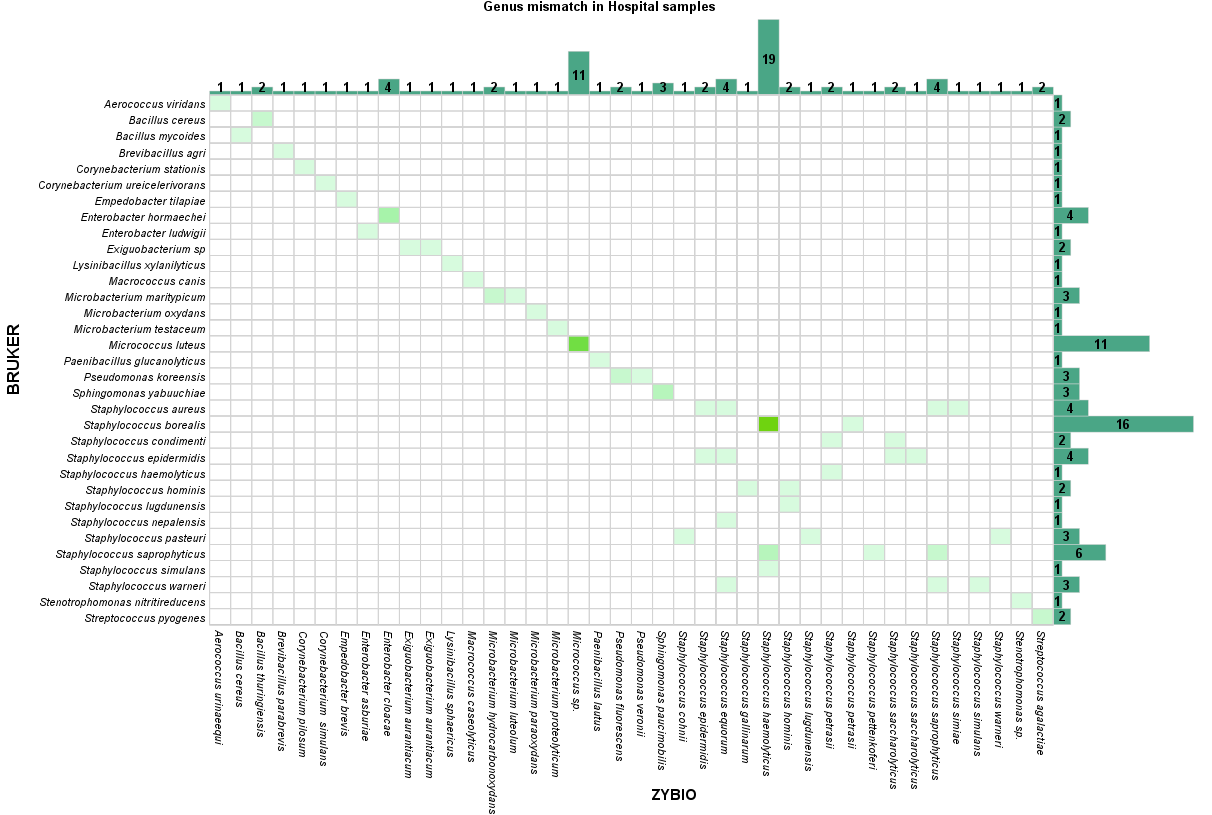


**c**

**d**


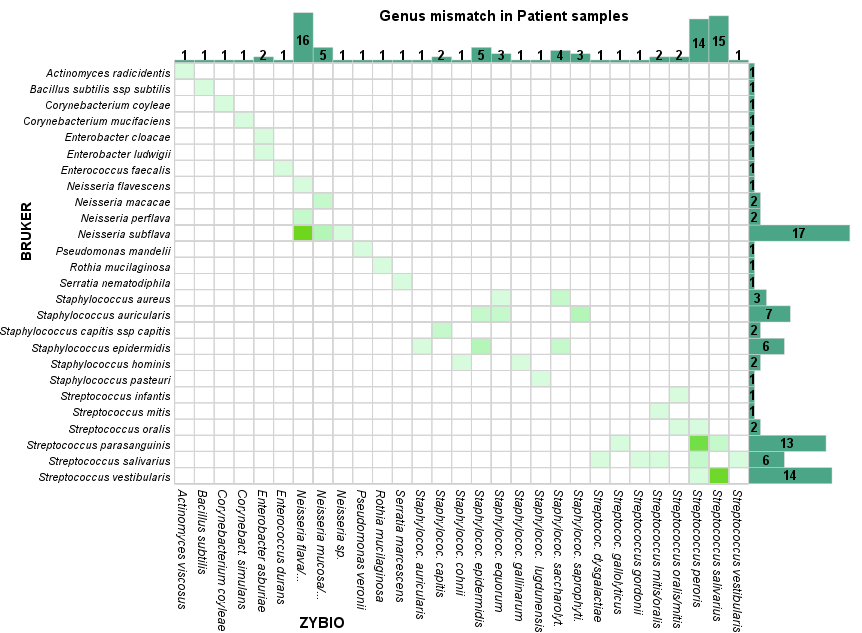


Supplementary Figure 2. All identified mismatches in BRUKER and ZYBIO identification. The matrix a-d summary of identified species- and genus- mismatch detected in patient samples (b,d) and hospital samples (a,c). The histogram indicates the number of identifications by two systems of MALDI. The colors inside matrix are correlated with the number of the appropriate bacteria species (from dark green- bacteria the most represented in the samples to light green- bacteria with low frequency in the samples).

| **NO** | **ID BRUKER** | **ID ZYBIO** | **ID 16S RNA NCBI Best match** | **Identified as** |
| --- | --- | --- | --- | --- |
| 1 | *Staphylo-coccus borealis* | *Staphylo-coccus haemolyticus* | *S. borealis* strain 51-48 [NR_181247] 99.79%, *S. haemolyticus* JCM 2416 [ NR_113345] 99.65% | *S. borealis/ haemolyticus* |
| 2 | *Bacillus pumilus* | *Bacillus lines* | *Bacillus pumilus* ATCC7061 [NR_043242] 100%, *Bacillus pumilus* NBRC12092 [NR_112637] 100% | *B. pumilus* |
| 3 | *Cytobacillus oceani-sediminis* | *Bacillus firmus (current Cytobacillus firmus)* | *Cytobacillus oceanisediminis* strain H2 [NR_117285] 98.71%, *Cytobacillus firmus* strain NBRC 15306 [NR_112635] 97.99% | *Cytobacillus oceanisediminis* |
| 4 | *Neisseria subflava* | *Neisseria flava/ flavescens/ subflava/ perflava* | *Neisseria perflava* strain Branham 7078 [NR_117694] 99.64%, *Neisseria flavescens* strain N 155 [NR_025892] 98.43% | *Neisseria perflava* |
| 5 | *Staphylo-coccus saprophyticus* | *Staphylo-coccus haemolyticus* | *Staphylococcus saprophyticus* subsp. saprophyticus ATCC 15305 = NCTC 7292 [NR_074999] 100%, *Staphylococcus pseudoxylosus* strain S04009 [NR_180150] 100% | *Staphylococcus saprophyticus/ pseudoxylosus* |
| 6 | *Streptococcus salivarius* | *Streptococcus vestibularis* | *Streptococcus salivarius* strain ATCC 7073 [NR_042776] 99.86%, *Streptococcus vestibularis* ATCC 49124 [NR_042777] 99.79% | *Streptococcus salivarius/ vestibularis* |
| 7 | *Neisseria macacae* | *Neisseria mucosa/ macacae/ sicca* | *Neisseria macacae* strain M-740 [NR_117701] 99.57%,  *Neisseria sicca strain* ATCC 29256 [NR_121688] 99.43%, strain N16 [NR_117696] 99.43% | *Neisseria macacae/ mucosa/ sicca* |
| 8 | *Streptococcus parasanguinis* | *Streptococcus peroris* | *Streptococcus parasanguinis* strain ATCC 15912 [NR_024842] 99.44%, *Streptococcus koreensis* strain KCOM 2890 [NR_165737] 98.39% | *Streptococcus parasanguinis* |
| 9 | *Raoultella ornithino-lytica* | *Klebsiella oxytoca* | *Raoultella ornithinolytica* strain JCM6096 [NR_114736] 99.79%, *Raoultella planticola* strain NBRC 14939 [NR_113701] 99.43% | *Raoultella ornithinolytica* |
| 10 | *Streptococcus vestibularis* | *Streptococcus salivarius* | *Streptococcus salivarius* strain ATCC 7073 [NR_042776] 99.86%, *Streptococcus vestibularis* strain ATCC 49124 [NR_042777] 99.79% | *Streptococcus salivarius/ vestibularis* |
| 11 | *Staphylo-coccus aureus* | *Staphylo-coccus equorum* | *Staphylococcus aureus* strain NBRC 100910 [NR_113956] 99.93%, *Staphylococcus aureus* strain ATCC 12600 [NR_115606] 99.93% | *Staphylococcus aureus* |
| 12 | *Bacillus cereus* | *Bacillus thuringiensis* | *Bacillus proteolyticus* strain MCCC 1A00365 [NR_157735] 100.00%, *Bacillus cereus* strain IAM 12605 [NR_152692] 99.93% | *Bacillus cereus group* |
| 13 | *Paenibacillus glucanolyticus* | *Paenibacillus lautus* | *Paenibacillus glucanolyticus* strain NBRC 15330 [NR_113748] 100.00%,  *Paenibacillus lautus* strain JCM 9073 [NR_040882] 98.22% | *Paenibacillus glucanolyticus* |
| 14 | *Coryne-bacterium casei* | *Coryne-bacterium striatum* | *Corynebacterium casei* strain LMG S-19264 [NR_122062] 99.93%, *Corynebacterium stationis* strain ATCC 14403 [NR_116558] 98.34% | *Corynebacterium casei* |

Supplementary Table 1. Identification results of bacteria with identification discrepancies using three methods: MALDI ToF MS Bruker, MALDI ToF MS Zybio, and 16S RNA sequencing. The last column shows the final classification of organisms based on concordance of results. The colors in the table reflect the identification levels achieved by the MALDI-TOF MS method: green indicates identification accurate to the species level (score >2.00), while yellow indicates identification limited to the genus level (score <2.00)
